# Land conversion and pesticide use degrade forage areas for honey bees in America’s beekeeping epicenter

**DOI:** 10.1371/journal.pone.0251043

**Published:** 2021-05-13

**Authors:** Dan J. Dixon, Haochi Zheng, Clint R. V. Otto

**Affiliations:** 1 Department of Earth System Science and Policy, University of North Dakota, Grand Forks, ND, United States of America; 2 Northern Prairie Wildlife Research Center, U.S. Geological Survey, Jamestown, ND, United States of America; Universitat Leipzig, GERMANY

## Abstract

A diverse range of threats have been associated with managed-bee declines globally. Recent increases of two known threats, land-use change and pesticide use, have resulted from agricultural expansion and intensification notably in the top honey-producing state in the United States: North Dakota. This study investigated the dual threat from land conversion and pesticide use surrounding ~14,000 registered apiaries in North Dakota from 2001 to 2014. We estimated the annual total insecticide use (kg) on major crops within 1.6 km of apiary sites. Of the eight insecticides quantified, six showed significant increasing trends over the time period. Specifically, applications of the newly established neonicotinoids Chlothianidin, Imidacloprid and Thiamethoxam, increased annually by 1329 kg, 686 kg, 795 kg, respectively. Also, the use of Chlorpyrifos, which was well-established in the state by 2001 and is highly toxic to honey bees, increased by ~8,800 kg annually from 6,500 kg in 2001 to 115,000 kg in 2014 on corn, soybeans and wheat. We further evaluated the relative quality changes of natural/semi-natural land covers surrounding apiaries in 2006, 2010 and 2014, a period of significant increases in cropland area. In areas surrounding apiaries, we observed changes in multiple indices of forage quality that reflect the deteriorating landscape surrounding registered apiary sites due to land-use change and pesticide-use increases. Overall, our results suggest that the application of foliar-applied insecticides, including pyrethroids and one organophosphate, increased surrounding apiaries when the use of neonicotinoid seed treatments surged and the area for producing corn and soybeans expanded. Spatially, these threats were most pronounced in southeastern North Dakota, a region hosting a high density of apiary sites that has recently experienced corn and soybean expansion. Our results highlight the value of natural and semi-natural land covers as sources of pollinator forage and refugia for bees against pesticide exposure. Our study provides insights for targeting conservation efforts to improve forage quality benefiting managed pollinators.

## 1. Introduction

Approximately 70% of the world’s flowering plants and one-third of total food consumption in the United States (U.S.) depend upon pollinators [1, 2]. However, since 2013, beekeepers in the U.S. have reported average annual colony losses in excess of 30% [3], and wild-bee abundance has declined in many parts of the world [4]. Pollinator declines have been attributed to multiple factors, often acting individually or synergistically, including insect pests and diseases, pesticide exposure, and forage declines [5]. Of these threats, habitat loss in the form of grassland-to-cropland conversion can both reduce forage area and increase the likelihood of pesticide exposure [5–7].

In the Great Plains region of the U.S., temperate grasslands are tremendously important to the commercial beekeeping industry and honey bee (*Apis* mellifera) colony health [6, 8, 9]. Flowers blooming on grasslands provide pollen and nectar that honey bees need to complete their lifecycle and produce honey [6]. Diverse floral diets are positively correlated with improved health metrics including brood area [10] and immune responses [11]. Diets lacking in pollen abundance or quality lead to decreased colony size and the overall number of bees [10, 12]. The area of grassland surrounding apiaries is positively related to honey bee colony size [9], colony survival [13], and beebread protein content [12]. Given the positive relationship between grassland and bee health, it is not surprising that U.S. commercial beekeepers tend to choose apiary sites with larger areas of grassland or other uncultivated landcovers [8].

Today, beekeepers operate within a complex array of grasslands and agricultural lands; therefore, honey bees are exposed to a wide range of insecticides, fungicides, and herbicides [14]. Colony side-effects of sub-lethal pesticide exposure include delayed adult development [15], reduced brood comb longevity [15], and increased likelihood of being infected with the parasite *Nosema ceranae* [16, 17]. For individual bees, researchers have noted a variety of motor, memory, and behavioral effects [18]. For example, sub-lethal insecticide exposure to foraging bees may exacerbate the negative impacts of the parasitic mite *Varroa destructor* by reducing flight capacity [19]. The sources of these pesticides can be highly diverse considering the myriad of crops, pests, and management practices surrounding apiaries [20]. While agricultural fields can act as a direct source of contamination, research suggests adjacent non-agricultural lands (e.g. grasslands or herbaceous wetlands) can also act as a sink for pesticide contamination via drift and systemic uptake [21]. This key exposure route highlights the importance of understanding the spatial relations among cropland, pesticide use, and grasslands surrounding apiary sites.

The Northern Great Plains (NGP) region of the U.S. provides an ideal landscape for investigating the dual threat of forage loss and pesticide exposure on pollinators. The NGP landscape offers sustainable forage for bees with diverse floral resources over a long blooming season supported by abundant grassland ecosystems and thousands of wetlands [6, 22]. Along with an ideal summer climate, the region attracts commercial beekeepers from across the U.S. who transport over one million colonies to the NGP each summer for honey production and to allow their colonies to recover from the stresses of performing migratory crop pollination services [6, 23]. After the summer, colonies are transported to pollinate a range of fruits, vegetables, seeds and nuts along the Pacific Coast, a service valued at $11.6 billion USD annually in the U.S. [24]. However, in recent years, the NGP has experienced agricultural intensification, cropland expansion, and ephemeral wetlands and mixed and tallgrass prairie losses; some of the most at-risk yet productive ecosystems in North America [25–27].

The land-use shift toward expansion of cropland and reduction in grasslands and other natural land-covers can affect pollinators through multiple pathways. First, the loss of grasslands decreases the availability of floral resources to meet pollinator nutritional requirements for sustaining colony health and productivity [9, 12, 13]. Second, increased cropland introduces the potential threats of direct and indirect pesticide exposure to foragers and the colony [5]. Evidence supporting the relationships between land-use, pesticides, and honey bee health is growing [20, 28], but few studies have quantified the dual impacts of pesticides and land-use change on landscape suitability for supporting honey bee colonies. Field-level studies with limited sample sizes often report pesticide residues observed in colonies or plant tissues surrounding hive sites [7, 29]. Broad-scale studies with limited spatial details have estimated the total insecticide use at the national level [30] or the toxicity of individual pesticides to honey bees at the county level [31]. However, no studies have yet investigated pesticide applications surrounding registered apiaries or their impacts on core foraging land covers such as non-cultivated grasslands and other natural land covers over a large area in a major commercial-beekeeping state.

In this study, we aimed to 1) quantify the total insecticide use on corn, soybeans and wheat within a radius of 1.6 km (1 mile) of 14,000 registered apiary sites in North Dakota from 2001 to 2014, and (2) evaluate relative changes in the quality of forage-land (i.e., grassland, wetlands, forest) impacted by insecticide applications on adjacent croplands in 2006, 2010 and 2014, a period representing intense grassland conversion [8]. By merging spatiotemporal land-use and pesticide data, we highlight the impact of this dual threat on grassland quantity and quality for supporting honey bees in North Dakota.

## 2. Materials and methods

### 2.1 Study region

North Dakota, the leading honey-producing state in the U.S. [22], was historically comprised of tall, mixed, and short grass prairie, from east to west, respectively; however, much of the native land cover has been converted to crop production, especially on the eastern side of the state. The primary three crops in North Dakota are soybeans (2.87 Mha), wheat (2.7 Mha) and corn (1.83 Mha) [32]. Other crops include barley, sugar beets, sunflower, alfalfa, canola, and other less common specialty crops [32]. North Dakota also contains an array of natural and semi-natural land covers including rangelands, grasslands, ephemeral wetlands and woody shrublands. Collectively, the ideal climate and heterogeneous land covers with diverse floral resources provide sufficient pasturing grounds for commercial honey bees over the growing season [6, 8].

### 2.2 Apiary registration

**Fig 1:**
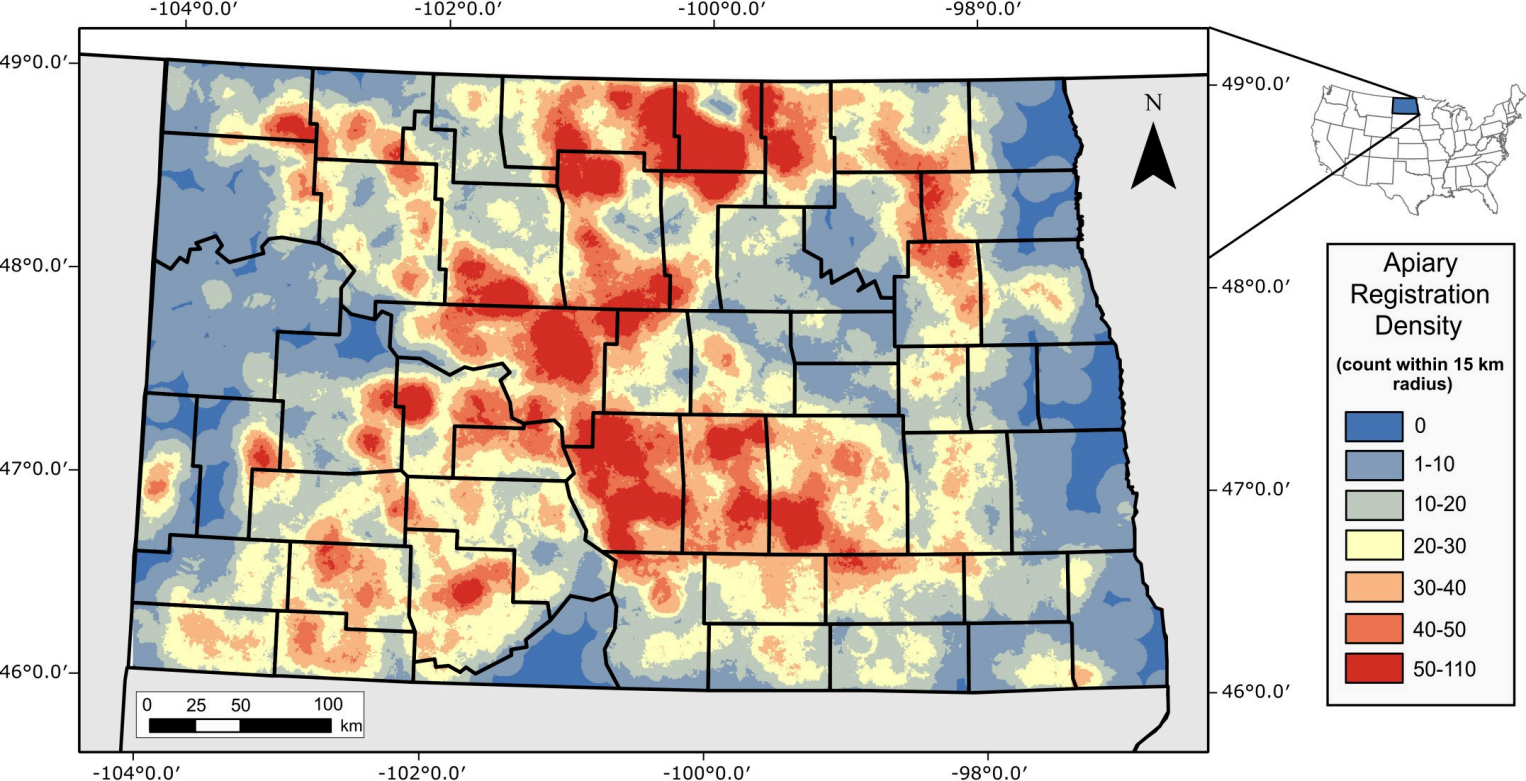
Density map of North Dakota registered apiaries (ND Department of Agriculture, 2014) from the year 2014. A 30x30 m raster grid was created where the value for each pixel represents the number of registered apiaries within a 15-km radius. Red pixels represent zones of the highest density. Black polygons distinguish North Dakota counties.

The North Dakota Department of Agriculture requires all apiary sites to be registered (https://www.nd.gov/ndda/plant-industries/apiary-honey-bees) at the quarter-section legal unit scale (one quarter section equals 65 ha) under the Public Land Survey System. This data set is regularly updated with the spatial location of each apiary site as well as the dates they were registered and cancelled. There were 13,477 apiary points registered in North Dakota in 2014, with the highest density in the center part of the state, less dense areas in the dry parts of western North Dakota and the intensely cropped Red River Valley in the east (Fig 1). Beekeepers normally stock around 50 colonies per apiary site in North Dakota [6]. As a conservative measure of pesticide applications within the area that are most likely to impact honey bees, we used the Geographic Information System (GIS) to place a 1.6 km buffer (≈314 ha) as the core flight area of foraging honey bees around each apiary point, following Otto et al. 2016 [8]. For each year of observation, we evaluated land-cover changes and pesticide application trends only within this buffer distance of each apiary site.

### 2.3 Land-cover data

To calculate annual pesticide use, we extracted cropland areas within each apiary buffer based on the US Department of Agriculture (USDA) Cropland Data Layer (CDL) (https://nassgeodata.gmu.edu/CropScape/), and we conducted trend analysis for pesticide applications from 2001 to 2014. We resampled the CDL layers to a 30 x 30 m spatial resolution and reclassified cover types into seven classifications: corn, soybeans, wheat, other cropland, wetland, forest, and grassland (S1 Table), accounting for 92% of the total area in ND. We further evaluated the habitat quality surrounding apiary sites based on the land covers in 2006, 2010, and 2014; 2006 to 2014 was a period of significant grassland conversion to cropland in North Dakota [8, 26]. We incorporated organic farms into the analysis by using an organic farms polygon shapefile obtained from the North Dakota Department of Agriculture (https://www.nd.gov/ndda/gis-maps) and assumed the insecticides of interest were not applied on these lands.

### 2.4 Spatiotemporal pesticide use

This research focused on insecticides and their use on primary crops in North Dakota. The selection of individual insecticides was based on two criteria. First, we selected active ingredients that were labeled as “high risk” or “moderate risk” according to a study of pesticide impacts on honey bees [33], which were derived from toxicity and residues commonly found in colonies. Second, insecticides were chosen if the US Geological Survey (USGS) EPest database (https://water.usgs.gov/nawqa/pnsp/) reported them as being used on corn, soybeans and / or wheat in North Dakota in at least seven of the fourteen years from 2001 to 2014. In total, eight insecticides met those criteria (Table 1), of which four (Chlorpyrifos, Thiamethoxam, Imidacloprid, and Clothianidin) were labeled as “high risk” and four (Bifenthrin, Cyfluthrin, Esfenvalerate and Cyhalothrin-lambda) were labeled as “moderate risk” [33]. It is important to note that USGS EPest database discontinued seed-treated estimates, which include neonicotinoids, for data after 2014.

**Table 1:** Insecticides, their class, and primary mode of application included in this study.

Compound	Class	Mode
Chlorpyrifos	Organophosphate	Spray
Esfenvalerate	Pyrethroid	Spray
Cyhalothrin-lambda	Pyrethroid	Spray
Bifenthrin	Pyrethroid	Spray
Cyfluthrin	Pyrethroid	Spray
Clothianidin	Neonicotinoid	Seed
Thiamethoxam	Neonicotinoid	Seed
Imidacloprid	Neonicotinoid	Seed

Pesticide data were gathered from the USGS EPest database which provides low and high estimates of total use (kg) of 423 unique active ingredients for each U.S. county from 1993–2018. EPest also provides low and high estimates of each pesticide applied on each crop type throughout the state. We first calculated a pesticide application rate, *AP*_*i*,*j*,*d*,*t*_, for each pesticide *i* on crop *j* in each Crop Reporting District (CRD, S6 Fig) *d* in year *t* using the following equation:

$${AP}_{i,j,d,t}=\frac{(\sum_{n=1}^{N_{d}}{Cnty}_{i,n,t})*{ratio}_{i,j,t}}{{Area}_{j,d,t}}$$ (Eq 1)

where *Cnty_i,n,t_* is the EPest low estimate of total application for county *n*, *N_d_* is the total number of counties in each district, *ratio_i,j,t_* is the Epest low estimate of pesticide-crop percentage, and *Area_j,d,t_* is the USDA NASS reported total planted area (in hectares) summed to the district. Thus, the total use of each pesticide *i* in year *t* is as follows:

$${TP}_{i,t}=\sum_{j,d}\left({AP}_{i,j,d,t}*{BF}_{j,d,t}\right)$$ (Eq 2)

where *BF_j,d,t_* represents the crop area within the 1.6-km apiary buffer. Given that many sites are located close to each other, we spatially combined the apiary buffers using the Dissolve tool in QGIS [34] to avoid double counting. Finally, we conducted a set of simple linear regressions in R [34] to explore the temporal trend of each pesticide used on the three main crops from 2001 to 2014. For each regression, the year was the independent variable, and the total use of each pesticide was the dependent variable.

### 2.5 Modeling degradation of adjacent natural covers

Focusing on the natural land covers (grasslands and herbaceous wetlands) within 1.6 km of each of the 13,477 registered apiaries in North Dakota, we modeled forage-land degradation from foliar applied insecticides (one organophosphate and three pyrethroids) in 2006, 2010 and 2014. Here, the application of insecticides on cropland was perceived as a potential threat to adjacent, natural, land covers because honey bees foraging on these lands have the potential to be exposed to these insecticides if they drift beyond the cropland. We used the Habitat Quality module from InVEST v3.4.2 [35], a spatial modeling tool that integrates land-cover information and related physical threats to quantify the changes in habitat across scenarios and time for a particular species or general biodiversity. The output of the model is a raster map in which pixels contain values ranging from 0 to 1, with 0 being lowest quality and 1 representing highest quality.

The inputs used were: 1) a baseline land-cover raster map (CDL); 2) layered threat rasters representing each insecticide-crop combination where each pixel was assigned a relative threat indicator based on risk quotients obtained with the pesticide application rate and LD 50 of the active ingredient (S1 Appendix; S2–S5 Figs); 3) a designated maximum distance as three pixels or 60 m within which 95% of spray material would be deposited exponentially [36]; and 4) a metric of how sensitive each land cover is to degradation. For simplicity, we assigned all cropland a habitat quality value of 0.0 and all “natural covers” a quality value of 1.0. Note that our main interest is the impact of pesticides on natural covers such as grassland and wetland; “bee-friendly” crops such as alfalfa, canola, and sunflower were assigned a value of 0 and thus not modeled in the current study.

### 2.6 Assessing InVEST model outputs

We designed two indices ranging from 0 to 1 to evaluate the InVEST output rasters for each apiary site: Quality Index (QI) and Degradation Index (DI), where QI scores of 1 represent high habitat quality and DI scores of 1 represent high degradation (lower quality). The QI for registered apiary site *s* in year *t* is defined as the average of the quality score for all pixels within the 1.6-km buffer:

$${QI}_{s,t}=\frac{\sum_{p}{{(I}_{p,s}*QI}_{p,t})}{9018}$$ (Eq 3)

*with I_p,s_* = 1 if pixel *p* is within the 1.6-km buffer of site *s*; *I_p,s_* = 0, otherwise. *QI_p,t_* indicates the InVEST model output on habitat quality between 0 and 1 for pixel *p*, and 9018 is the total number of pixels surrounding a site within a 1609-m radius. The QI measures the overall quality of an apiary site by considering both the available amount of natural land covers and the degree to which they were degraded due to pesticide threat layers. The DI calculates a ratio of quality changes that occurred on the natural land cover surrounding a site strictly due to pesticide threat, i.e., how much they deviate from “pesticide-free” status:

$${DI}_{s,t}=\frac{\sum_{g}\left(I_{g,s}*(1-{QI}_{g,t})\right)}{\sum_{g}I_{g,s}}$$ (Eq 4)

where *QI_g,t_* indicates the habitat quality score of the natural land-cover pixel g in year t *with I_g,s_* = 1 if pixel g is a natural land pixel within the 1.6-km buffer of site s; otherwise *I_g,s_* = 0. The difference between QI and DI is that the former evaluates the collective impacts of both pesticide application and the quantity changes of natural land cover to apiary sites while the latter isolates the impact of pesticides given the existing amount of natural lands. We averaged the scores across all apiary sites to evaluate the overall habitat-quality changes in 2006, 2010, and 2014. For map visualizations, all spatial polygons were obtained from the North Dakota GIS portal (https://www.gis.nd.gov) and maps were created using QGIS [34].

## 3. Results

### 3.1 Insecticide use around apiaries

**Fig 2:**
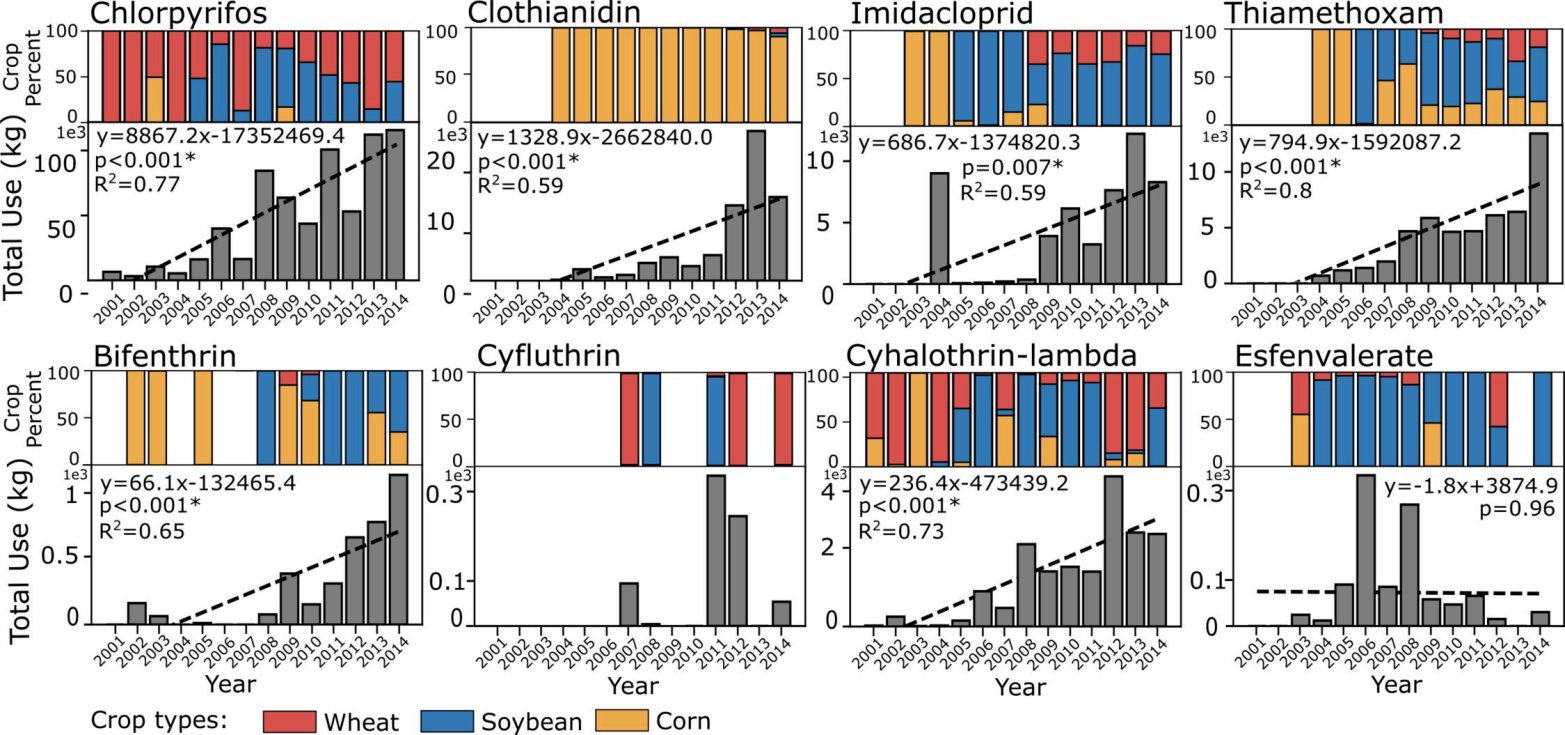
Eight insecticides average total use (kg) per site and their trends within 1.6 km of registered apiaries from 2001–2014. The upper panels show each insecticide’s use percent on wheat (red), soybeans (blue) and corn (red). Data points with zero uses were treated as missing values and were removed from each regression.

Of the eight insecticides we selected, six showed significant increasing total use trends from 2001–2014 within 1.6 km of registered apiary sites on corn, soybeans and wheat (Fig 2).

The newly introduced neonicotinoids Clothianidin, Imidacloprid, Thiamethoxam increased annually by 1329.9 kg, 686.7 kg, 795 kg, respectively, during the study period (Fig 2). Unexpectedly, Chlorpyrifos (organophosphate) and Cyahlothrin-lamba (pyrethroid), the two insecticides that were already well established by 2001, showed persistently increasing trends of 8,867.2 kg/year and 236.4 kg/year, respectively. The pyrethroid Esfenvalerate, on the other hand, reached a peak in 2006 but showed no significant linear trend over the study period (Fig 2).

The sources of insecticide use from the three crops varied over time. Chlorpyrifos was rarely used on wheat and corn from 2001 to 2004, but increased sharply after 2004 due to its use on soybeans and wheat (Fig 2). Bifenthrin and Cyhalothrin-lamba showed increasing use on soybeans in the second half of the study period (Fig 2). Clothianidin was sourced almost exclusively from corn, which reflects its primary use as a seed treatment. Imidacloprid was primarily used on soybeans while Thiamethoxam fluctuated among corn, soybeans and wheat (Fig 2).

### 3.2 InVEST model outputs: Foliar applied insecticides

**Fig 3:**
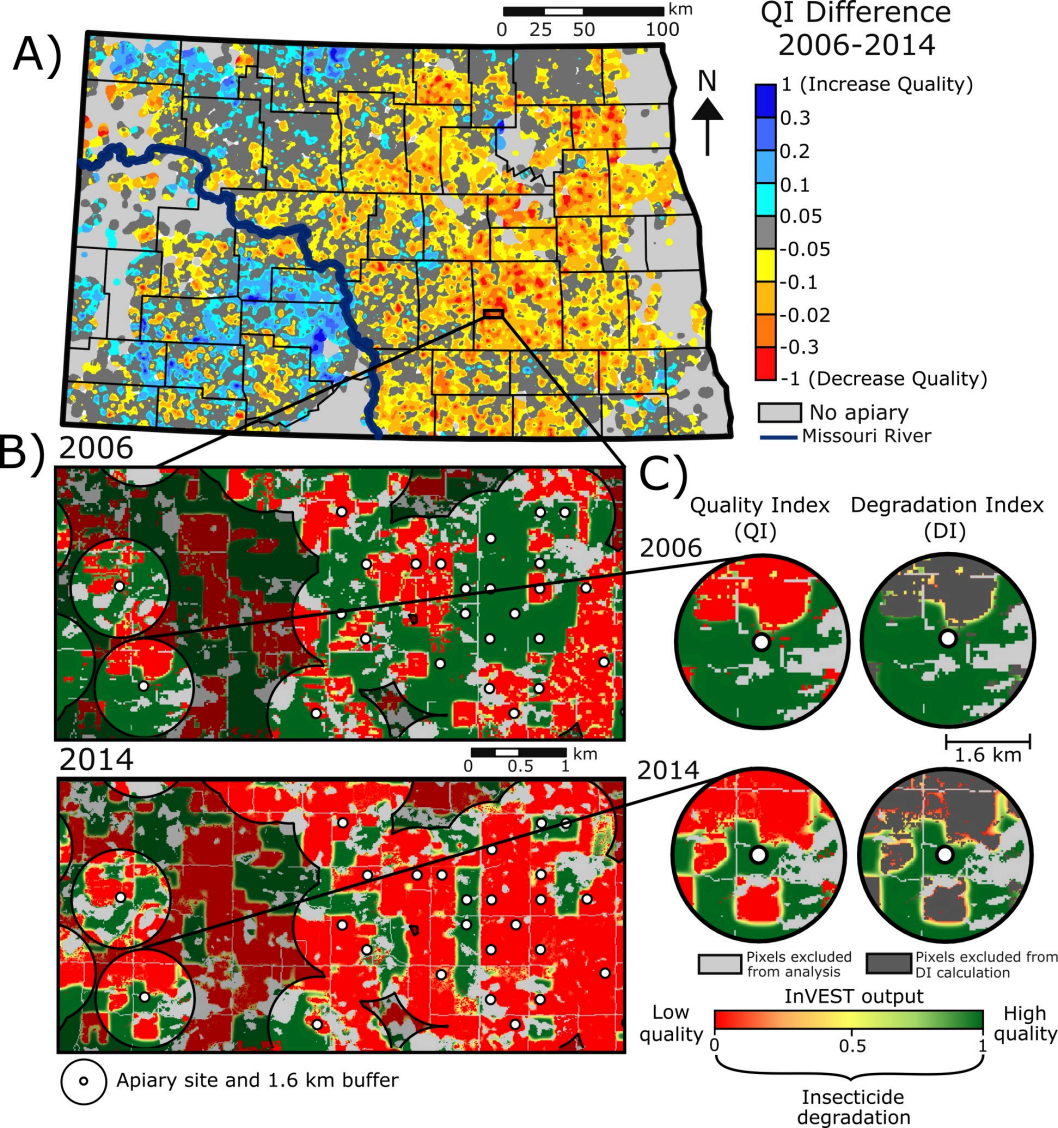
Difference in Quality Index (QI) scores from 2006–2014 surrounding North Dakota apiaries. A) The change in QI scores between 2006–2014 on lands within 1.6 km of registered apiary sites in North Dakota. Negative values (red) and positive values (blue) indicate decreasing and increasing QI scores, respectively; B) Example of changing QI from 2006–2014 in Stutsman County North Dakota, where grasslands hosting several apiary sites in 2006 were converted to cropland in 2014; C) Example of changes in QI and DI for individual site with red and green values indicating low and high quality habitat, respectively, from 2006 and 2014.

The mean Quality Index (QI) for all North Dakota apiaries, which represents habitat quality and degradation from insecticide use, increased slightly from 0.485 (± 0.0041; 95% confidence interval) in 2006 to 0.501 (± 0.0042) in 2010 and then decreased to 0.428 (± 0.0043) in 2014, a decrease of 11.75% from 2006 to 2014. Varying changes in apiary QI were evident throughout North Dakota comparing 2006 and 2014 (Fig 3). The habitat quality (QI) surrounding apiaries decreased most in central-eastern counties east of the Missouri River (Fig 3A). The conversion of large grassland parcels to cropland contributed to the largest decreases in QI scores (Fig 3A). Please see S6 Fig to visualize the distribution of QI scores in 2006 and 2014.

This phenomenon is especially highlighted in Stutsman County, southeastern North Dakota, where grasslands with marginal soils were converted to corn/soybean rotation (Fig 3B). In contrast, the habitat quality (QI) scores improved in the western part of the state where wheat, sunflower and barley have been replaced by herbaceous wetland and grass/pasture since 2006 (Fig 3A).

Pesticide degradation also caused noticeable differences in the remaining grassland quality alongside cropland/natural land-cover edges (Fig 3B and 3C). This is evident in the increased number of orange/yellow pixels, mainly on the boundary of natural land covers resulting from increased insecticide use, fragmentation, and increased area of adjacent cropland (Fig 3B and 3C).

**Fig 4:**
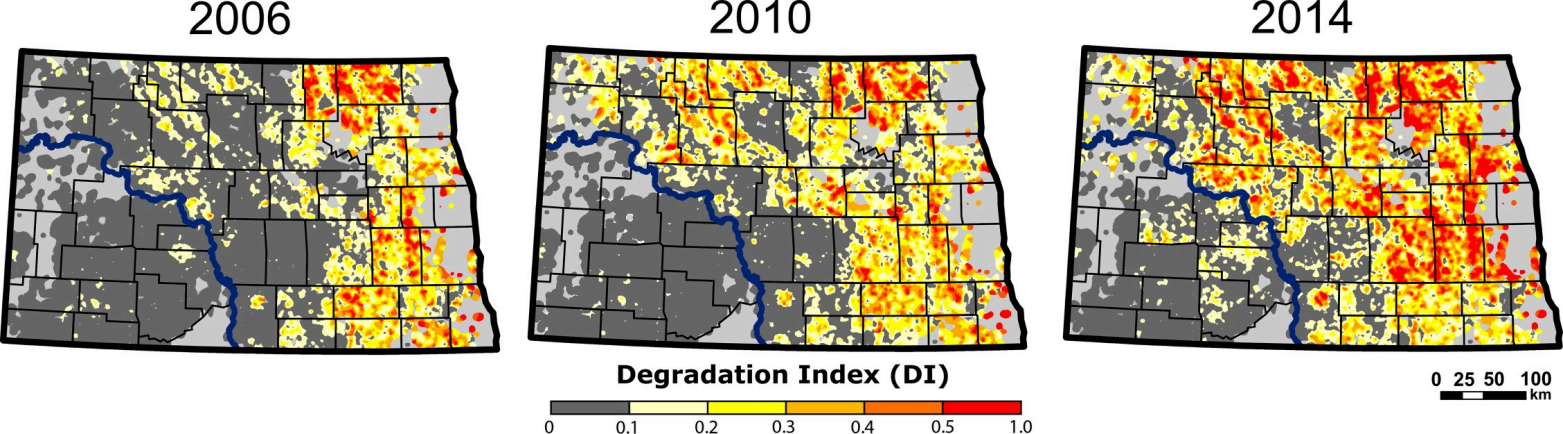
Degradation Index (DI) outputs from InVEST model within apiary site 1.6-km buffers. Pixels that have the high, low, and minimal (ranging from 0 to 1.0 changes are represented as red, yellow, and dark grey, respectively. The light grey regions did not have registered apiaries.

The degradation specifically from insecticide use, as captured by the Degradation Index (DI), was highest in 2014 with a mean of 0.19 (± 0.002), which is a 95.3% increase from 2006 (mean = 0.0973 (± 0.003)). The spatial distribution of insecticide degradation (DI) revealed that degradation of natural land covers surrounding apiary sites was concentrated in the eastern portion of North Dakota (Fig 4). From 2006 to 2014, degradation gradually moved westward to impact the majority of apiaries east of the Missouri River (Fig 4). Please see S6 Fig to visualize the distribution of DI scores in 2006 and 2014.

## 4. Discussion

Increased use of pesticides and land-use change have been implicated in bee declines globally [5]. Our study attempted to model the joint effects of land-use change and insecticide use on landscape suitability for supporting honey bees. While other studies have acknowledged the negative effect of multiple stressors on pollinators, it is often challenging to show how multiple stressors interact over space and time to affect the forage landscape for bees. Our novel approach integrated data at multiple scales and partitioned the local-scale pesticide risk (DI) from the large-scale changes of land-use (QI) within close proximity to commercial apiaries in North Dakota. Decreases in the Quality Index scores that we observed are supported by past research describing significant loss of grasslands and wetlands that occurred from 2006 to 2014 in our region [26, 37] and increasing pesticide use at a national level since 2000 [30, 31]. While our research lacks an experimental approach demonstrating the direct effects of land-use change and insecticide applications on honey bee colonies, it does provide a spatially robust depiction how these well-known threats to bees are becoming more common in the top honey-producing state in the U.S. [22]. This is disconcerting considering the number of registered honey bees colonies brought to North Dakota increased from 280,000 in 2001 to 490,000 in 2014 [38, 39]. Thus, the North Dakota landscape is becoming less conducive to supporting honey bees at a time when the number of honey bee colonies on the landscape is rapidly increasing.

The period of 2001–2014 represents a critical time of neonicotinoid expansion and continued, traditional-insecticide use on lands occupied by U.S. commercial beekeepers. While we expected neonicotinoid use in North Dakota to mirror national trends [30], sharp increases in Chlorpyrifos, Cyhalothrin-lambda and Bifenthrin estimated in our study suggest neonicotinoids are not simply replacing other classes of insecticides, but are being used in addition to more traditional compounds. Increased use of both neonicotinoids and more traditional, foliar applied chemicals means honey bees are faced with multiple insecticide exposure routes throughout the growing season [20, 21]. Some commercial beekeepers have delayed transportation of their honey bees to North Dakota during the spring to avoid the corn and soybean-planting season when risk of neonicotinoid exposure is greatest [40]. The delayed arrival by beekeepers means they must keep honey bee colonies in holding yards elsewhere in the US, feed their bees artificial supplements, and forgo early-season honey production, all of which can incur significant revenue loss to beekeepers. It is unclear whether the trends we observed for neonicotinoids have continued beyond 2014, as the USGS Pesticide National Synthesis Project discontinued tracking seed-treated neonicotinoids for data after 2014 (https://water.usgs.gov/nawqa/pnsp/usage/maps/).

The increased use of pyrethroids and Chlorpyrifos we estimated around North Dakota apiaries may be attributable to the spread of soybean aphids (*Aphis glycines*) during our study period. Soybean aphids spread across 22 states in the 2000’s while arriving in eastern North Dakota in 2001 and 2002 [41, 42]. Because neonicotinoid seed treatments targeting early season pests were ineffective in controlling the peak of aphid abundance, foliar treatment and re-treatment have been implemented later in the growing season [30, 43–45]. While management alternatives to aphid infestations do exist such as biocontrol [46], current Integrated Pest Management schemes (IPM) recommend spray-based insecticides such as pyrethroids and organophosphates [47] which are highly toxic to honey bees [33]. As beekeepers increasingly operate near soybean fields [8], this exposure route underscores the importance of additional IPM guidelines which recommend timely and precise applications only when economic thresholds are reached [48].

Our results reinforce existing literature regarding the important role played by grasslands and wetlands in North Dakota as beneficial forage resources [9, 49]. We further highlight the critical function provided by these natural land covers on mitigating pesticide exposure by providing refuge from cropland where pesticide drift and debris originate. Recent studies have shown that wildflower strips adjacent to cropland can act as pesticide sinks and attract foraging bees [5, 29]. The variation of our Degradation Index indicates that not all natural land covers provide equal resource quality because resource degradation occurs when pesticide drift interacts with neighboring wildflowers. Thus, our research highlights the importance of large, continuous grassland patches in providing safe foraging areas for bees in agricultural landscapes. Although small forage areas along field margins are also important to bees, these areas present addition insecticide exposure routes if the adjacent cropland is being actively treated.

One of the limitations of spatially modeling the impacts of insecticide use on landscape suitability for honey bees is that we were unable to take into account modes and timing of application, and environmental factors that could affect bee exposure. For example, we were unable to account for how fine-scale land features such as hedgerows may mitigate spray drift into adjacent grasslands [50], or how timing of insecticide applications during periods when bees are unlikely to be foraging may reduce exposure risk [51]. Insecticide applicators can elect to plant treated seed or spray foliar chemicals when wind conditions would minimize drift. These field-level factors are challenging to incorporate into landscape models but will have significant impact on insecticide exposure risk. Furthermore, our pesticide application rates are calculated at the district scale, and therefore are a function of multiple factors including the number of users and how much they applied, all of which are derived from proprietary surveys and aggregated which are subjected to potential sampling issues. Consequently, we do not attempt to model the true environmental fate of pesticides within or around individual colonies.

Given the large-scale nature of our study, we were forced to make several assumptions relating to the preexisting quality of land covers, the specific pesticides and crops included, and the behavior of pesticides in the environment. We first assumed that all natural land covers provide equal resources for pollinators. This assumption may not hold given the climate differences across the state, land management practices and natural variation in grassland quality. However, we currently lack the tools to describe such land covers based on the quantity and or quality of forbs they contain at the landscape scale. We also acknowledge that our models are representative of the active ingredients and crops we selected. Our model did not include specialty “bee-friendly” crops such as alfalfa, sunflower and canola because their spatial accuracy in the CDL is less consistent than primary crops, therefore unsuitable for conducting analysis at pixel scale over a long-term period [6]. These bee-friendly crops undoubtably act as a source of nutrition for honey bee colonies but also present risk to foragers if the crops have been treated with insecticides [52]. Modeling these more complicated interactions between bee-friendly crops and insecticide applications was beyond the scope of this work but is crucial for further defining and understanding pesticide exposure routes to bees in agroecosystems. Future research investigating changes in landscape suitability for honey bees or wild bees should consider including “bee-friendly” crops and crop-specific insecticide applications. This research could be further strengthened by additional field and laboratory research that quantifies how insecticide usage to “bee-friendly” crop fields affects the health of foraging bees.

## 5. Conclusion

This research merges two key threats impacting honey bee declines: insecticide use and land-use change to understand landscape degradation in North Dakota for supporting honey bee colonies. During the period 2001–2014, others have reported drastic grassland-to-cropland conversion [8, 26]. However, we contribute to the literature by quantifying the additional component of pesticide use over time and space surrounding bee yards. Both neonicotinoids and traditional foliar-applied insecticides increased during this time suggesting both expansion of cropland area and intensification of agricultural practices that affect honey bees. Our research underscores the value provided by grasslands and other natural areas for supporting commercial honey bees and the pollination services they provide.

## Supporting information

S1 Fig Nine Crop Reporting Districts (CRDs) in North Dakota and counties showing spatial scale of pesticide application rates. CRDs abbreviated with directions for North, South, East, West, and Central.
(JPG)

S2 Fig Threat rasters of BIFENTHRIN application in North Dakota crop reporting districts. (TIF)

S3 Fig Threat rasters of CYHALOTHRIN-LAMBDA application in North Dakota crop reporting districts. (TIF)

S4 Fig Threat rasters of CHLORPYRIFOS application in North Dakota crop reporting districts. (TIF)

S5 Fig Threat rasters of ESFENVALERATE application in North Dakota crop reporting districts. (TIF)

S6 Fig Density plots of InVEST model outputs. Shows the distribution of Quality Index (A) and Degradation Index (B) for all registered apiaries (N = 13,477) in 2006 (blue) and 2014 (orange). Mean values are also shown with 95% confidence intervals.
(JPEG)

S1 Table Land cover reclassifications for rasters used in pesticide quantification and threat modelling. Cropland Data Layer original values were reclassified to one of eight classes: NA = -999, other crops = 254, corn = 1, wheat = 3, soybeans = 5, forest = 63, wetland = 83, grassland = 176.
(PDF)

S2 Table Spray applied risk quotients used as inputs for Bee-REX oral and tactile RQ calculation. Values were obtained from Sanchez-Bayo and Goka (2014).
(PDF)

S1 Appendix Threat layer inputs to InVest model. (PDF)
