# Supplementary figures and images for: Land conversion and pesticide use degrade forage areas for honey bees in America’s beekeeping epicenter
Source: PLoS One. 2021 May 13;16(5):e0251043. doi: 10.1371/journal.pone.0251043 (PMC8118293; doi:10.1371/journal.pone.0251043)

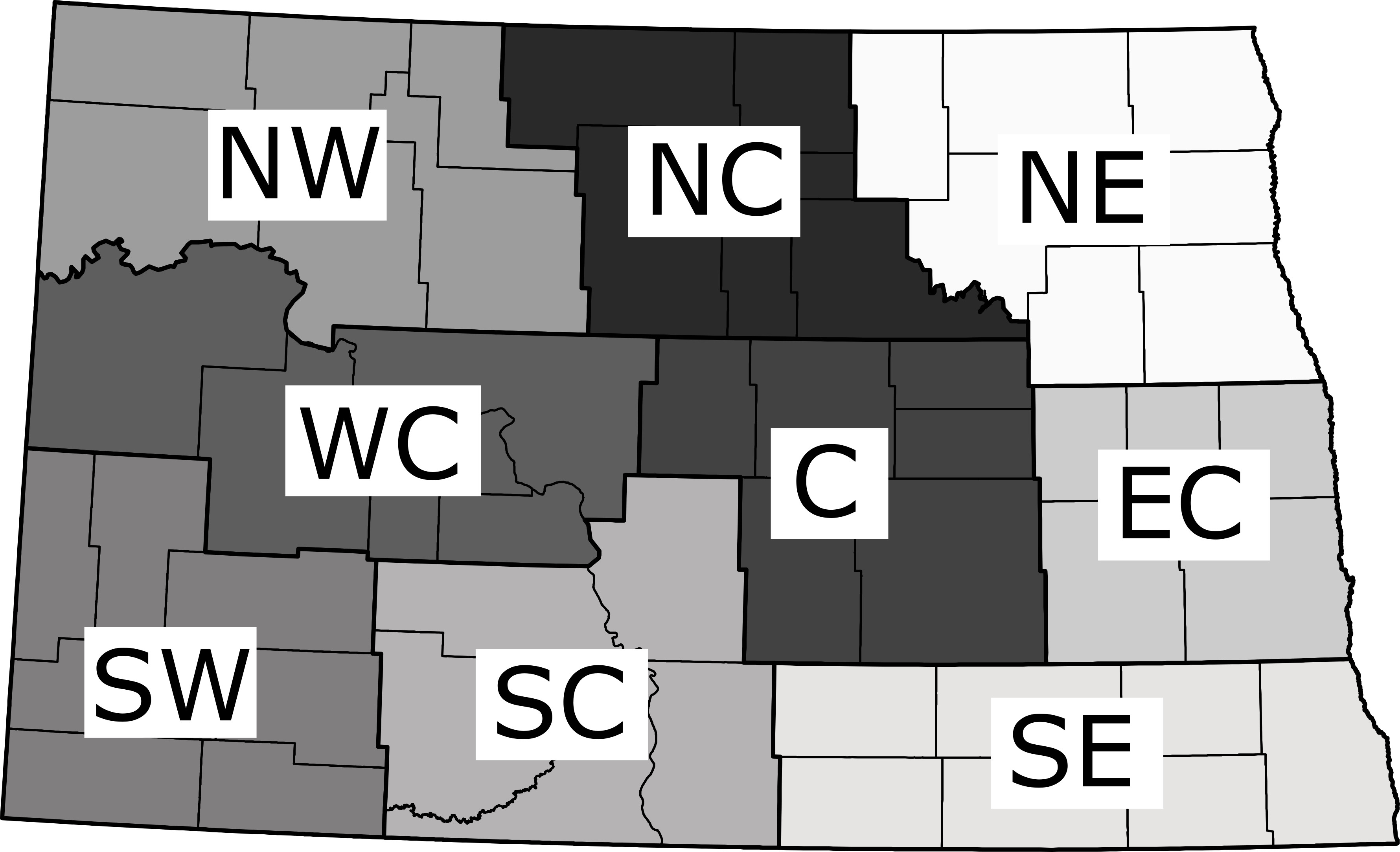

Supplement: S1 Fig — CRDs abbreviated with directions for North, South, East, West, and Central. (JPG) [file pone.0251043.s001.jpg]

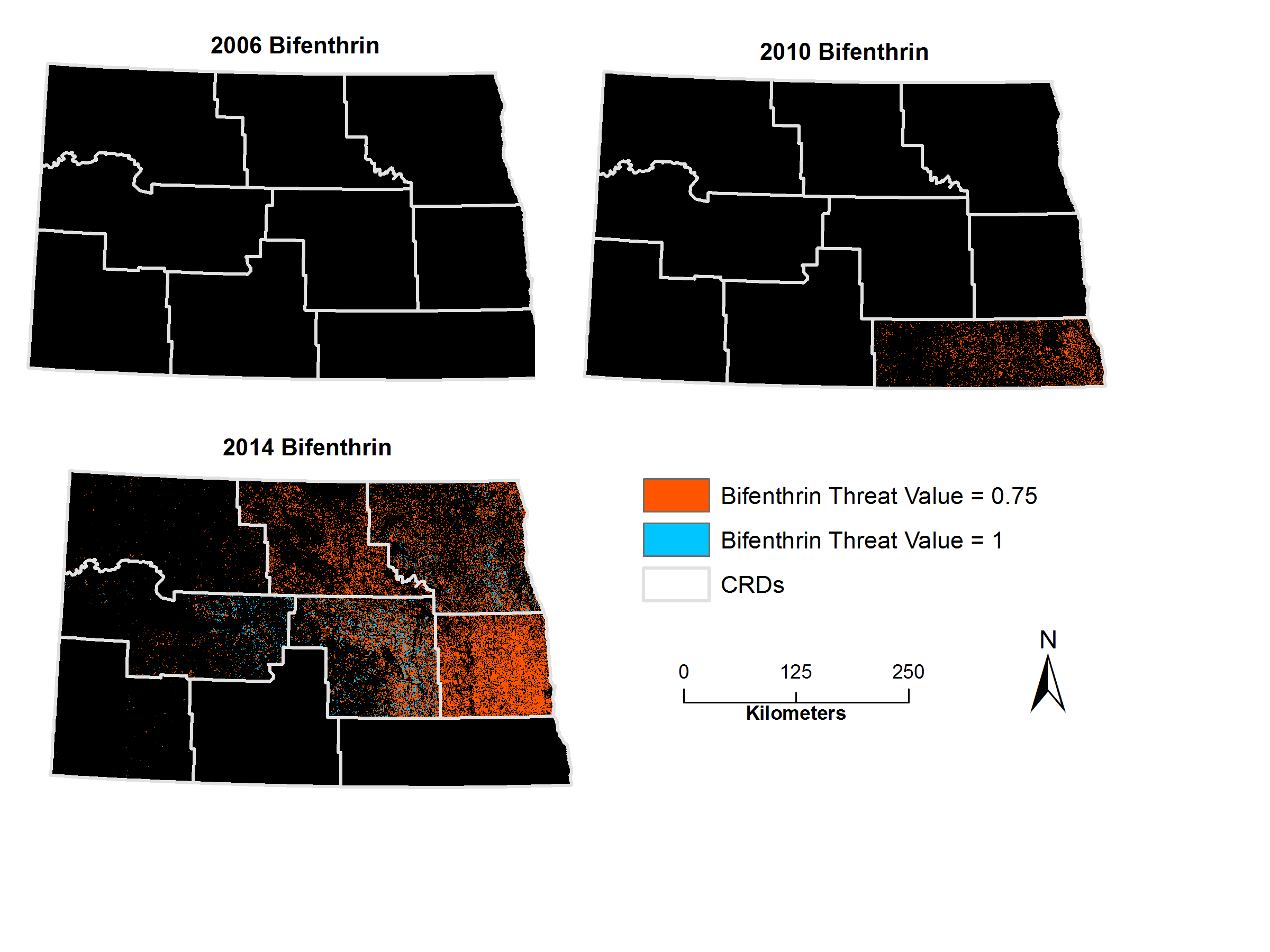

Supplement: S2 Fig — (TIF) [file pone.0251043.s002.tif]

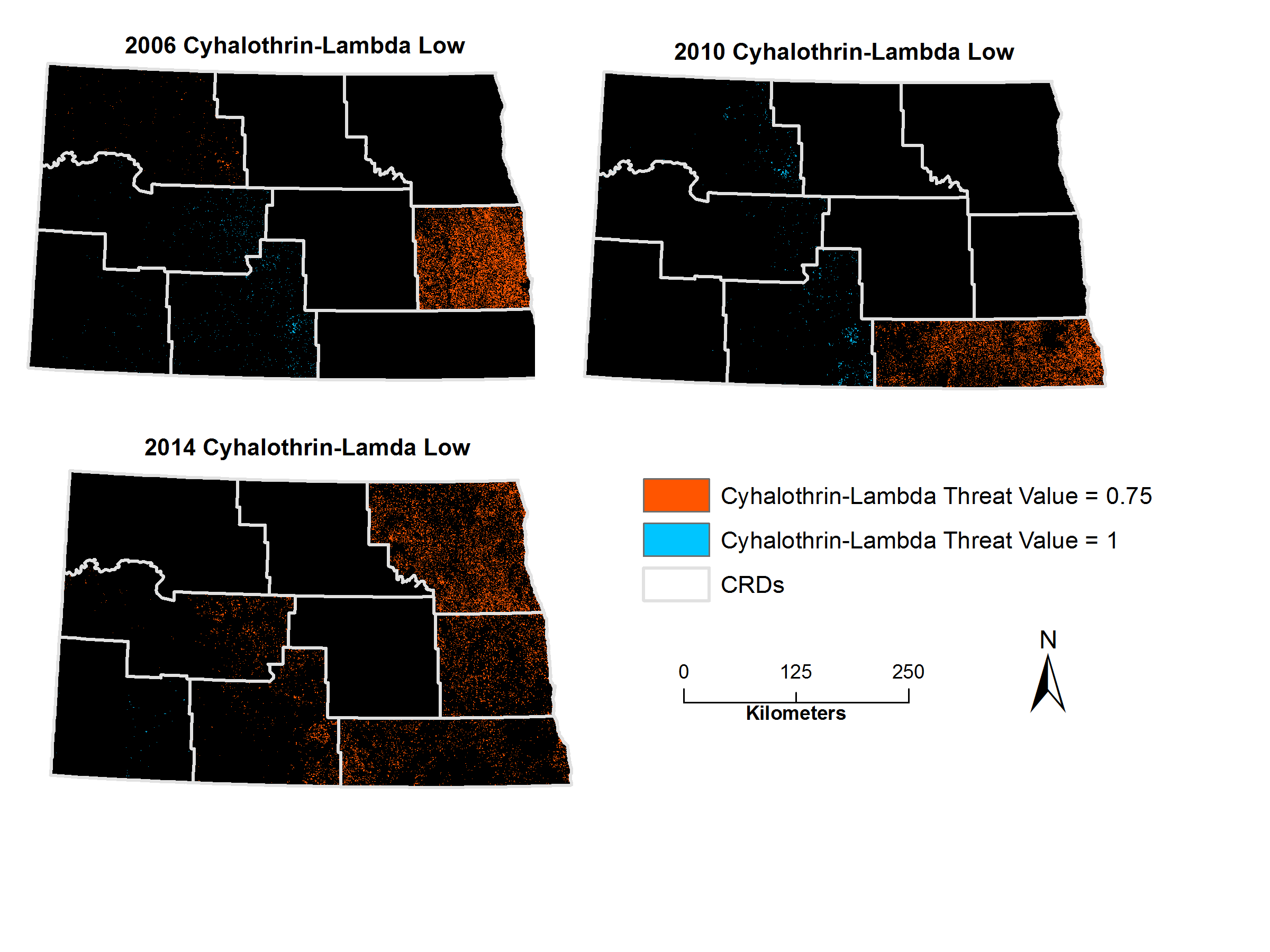

Supplement: S3 Fig — (TIF) [file pone.0251043.s003.tif]

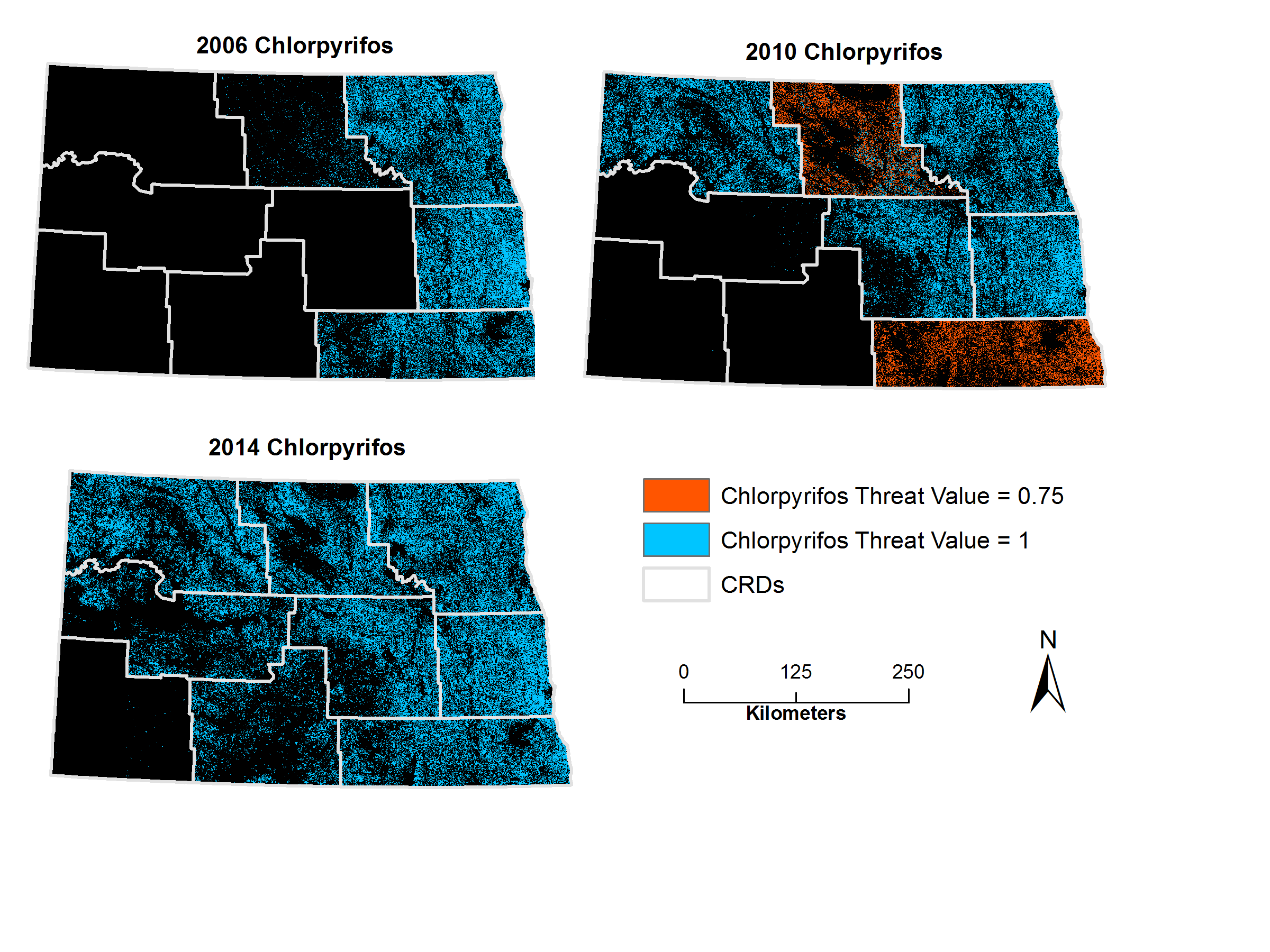

Supplement: S4 Fig — (TIF) [file pone.0251043.s004.tif]

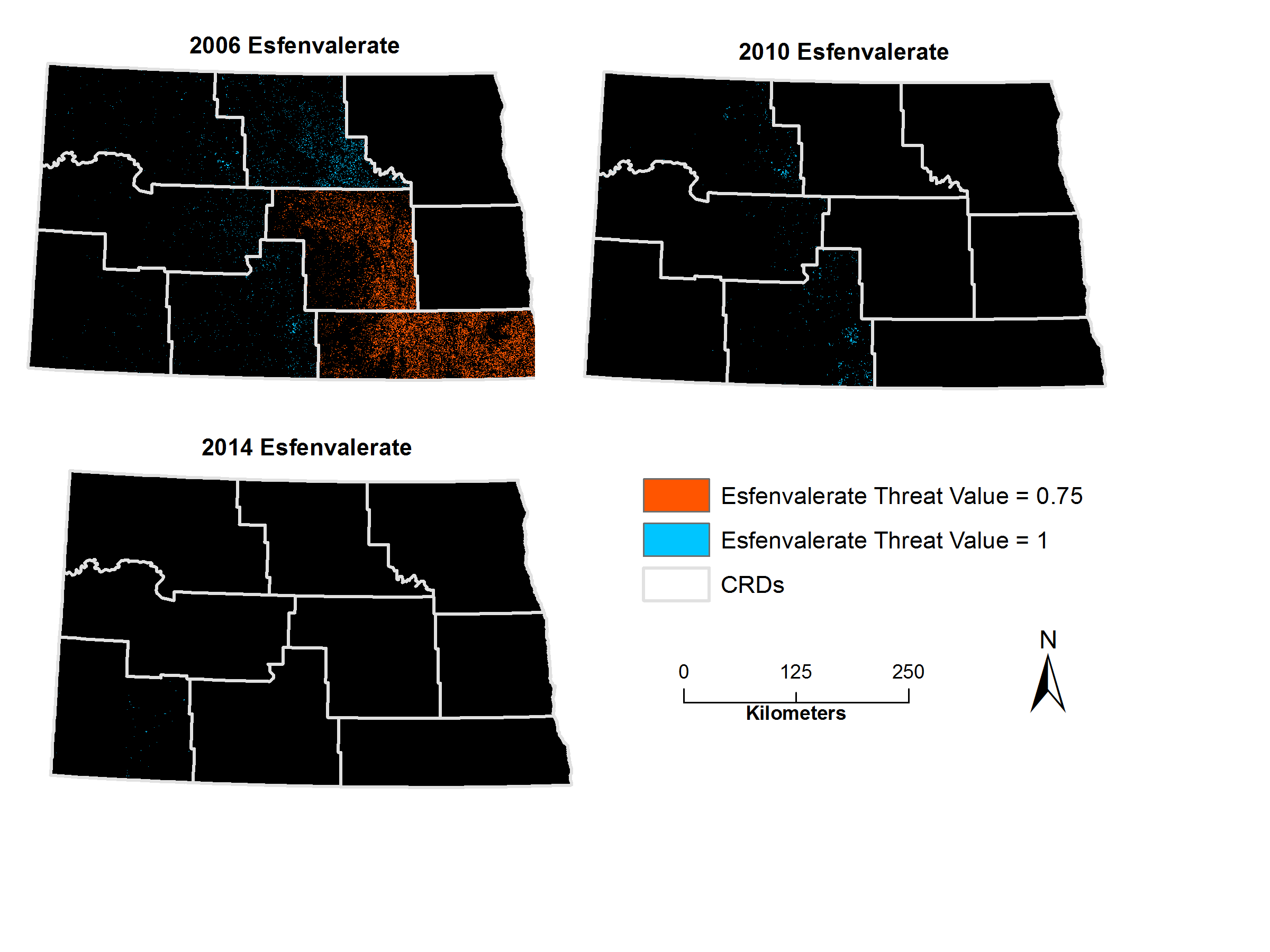

Supplement: S5 Fig — (TIF) [file pone.0251043.s005.tif]

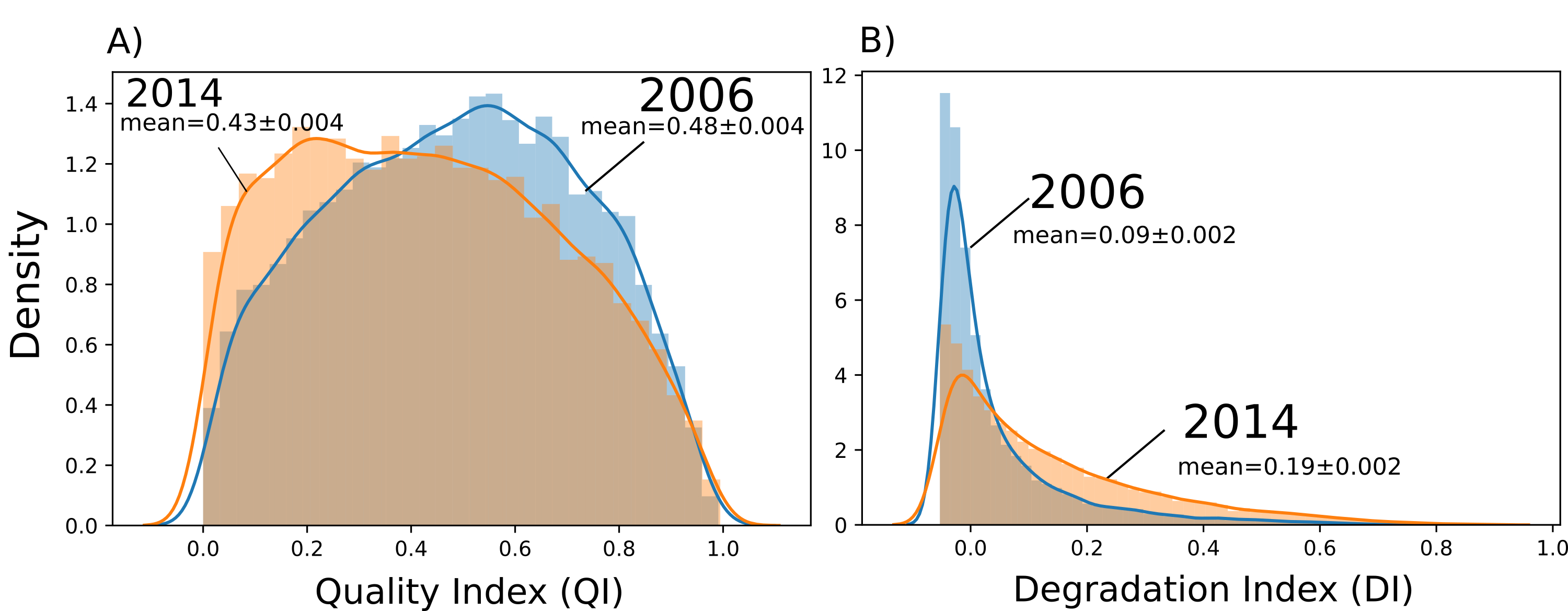

Supplement: S6 Fig — Shows the distribution of Quality Index (A) and Degradation Index (B) for all registered apiaries (N = 13,477) in 2006 (blue) and 2014 (orange). Mean values are also shown with 95% confidence intervals. (JPEG) [file pone.0251043.s006.jpeg]
