# Supplementary material for: Land conversion and pesticide use degrade forage areas for honey bees in America’s beekeeping epicenter
Source: PLoS One. 2021 May 13;16(5):e0251043. doi: 10.1371/journal.pone.0251043 (PMC8118293; doi:10.1371/journal.pone.0251043)
Supplement: S1 Table — Cropland Data Layer original values were reclassified to one of eight classes: NA = -999, other crops = 254, corn = 1, wheat = 3, soybeans = 5, forest = 63, wetland = 83, grassland = 176. (PDF) [file pone.0251043.s007.pdf]

Original Description	Original value	Reclassify value
Background	0	-999
Sorghum	4	254
SweetCorn	12	254
PopOrdCorn	13	254
Barley	21	254
OtherSmallGrains	25	254
DblWinWhtS	26	254
Rye	27	254
Oats	28	254
Millet	29	254
Speltz	30	254
Flaxseed	32	254
Safflower	33	254
Mustard	35	254
Camelina	38	254
Buckwheat	39	254
Sugarbeets	41	254
DryBeans	42	254
Potatoes	43	254
OtherCrops	44	254
MiscFruitsVeg	47	254
Watermelons	48	254
Onions	49	254
Lentils	52	254
Peas	53	254
Herbs	57	254
Barren	65	254
Apples	68	254
clouds/nodata	81	-999

Developed	82	-999
Water	83	-999
OpenWater	111	-999
DevelopedOpen	121	-999
DevelopedLow	122	-999
DevelopedMed	123	-999
DevelopedHigh	124	-999
Barren	131	-999
Triticale	205	254
Vetch	224	254
DblWinWhtC	225	254
DblOatsCorn	226	254
Pumpkins	229	254
DblBarleySor	235	254
DblWinWhtS	236	254
DblSoybeans	240	254
DblCornSoybeans	241	254
Radishes	246	254
Turnips	247	254
Corn	1	1
DurumWheat	22	3
SpringWheat	23	3
WinterWheat	24	3
Soybeans	5	5
Sunflower	6	254
Canola	31	254
RapeSeed	34	254
Alfalfa	36	254
Forest	63	63
DeciduousForest	141	63
EvergreenForest	142	63

MixedForest	143	63
Wetlands	87	83
WoodyWetlands	190	83
HerbaceousWetlands	195	83
OtherHay	37	176
CloverWildflowers	58	176
Sod	59	176
Switchgrass	60	176
FallowIdle	61	176
GrassPasture	62	176
Shrubland	152	176
GrasslandHerbaceous	171	176
GrassPasture	176	176
PastureHay	181	176
