# Supplementary material for: Land conversion and pesticide use degrade forage areas for honey bees in America’s beekeeping epicenter
Source: PLoS One. 2021 May 13;16(5):e0251043. doi: 10.1371/journal.pone.0251043 (PMC8118293; doi:10.1371/journal.pone.0251043)
Supplement: S2 Table — Values were obtained from Sanchez-Bayo and Goka (2014). (PDF) [file pone.0251043.s008.pdf]

Compound	LD50 Oral	LD50 Tactile
Chlorpyrifos	0.24	0.07
Esfenvalerate	0.21	0.03
Cyhalothrin-lambda	0.97	0.05
Bifenthrin	0.2	0.01
Cyfluthrin	0.05	0.03
