# Supplementary material for: Land conversion and pesticide use degrade forage areas for honey bees in America’s beekeeping epicenter
Source: PLoS One. 2021 May 13;16(5):e0251043. doi: 10.1371/journal.pone.0251043 (PMC8118293; doi:10.1371/journal.pone.0251043)
Supplement: S1 Appendix — (PDF) [file pone.0251043.s009.pdf]

S1 Appendix. Threat layer inputs to InVest model

Layered threat rasters represent the relative threat of foliar applications to honey bees. The relative threat of a foliar application to foraging honey bees depends on not only its application rate, but other multiple factors like the LD50 of the active ingredient. To measure and be able to compare relative toxicities across different pesticides and crops, we normalized the estimated application rates based on risk quotients using an equation from the U.S. Environmental Protection Agency BeeREX v.1.0, a terrestrial model for assessing risk of pesticide applications on individual foraging honey bees (www.epa.gov/pollinator-protection/pollinator-risk-assessment-guidance). We averaged oral and tactile risk quotients (RQ) based on each insecticide's application rate and its LD50 (Sanchez-Bayo and Goka, 2014, Table 1 SI) and then compared those values to a level of concern (LOC) of 0.4 suggested by BeeREX. We rescaled the RQ numbers and compiled the threat raster for each pesticide-crop-year combination to be input in the InVEST model. Ultimately, a value of 1 (highest threat) was assign to CRC-crop-pesticide combinations where risk quotients exceeded the LOC. For RQ values between 0.1 and 0.4, these pesticide applications were assigned a risk score of 0.75. Additionally, all cropland including "other crops" received a constant pesticide threat value of 0.5 to account for other pesticides and crops not included in this analysis. See S2-5 Figs for visualization of pesticide threat layers used as inputs.
